# The influence of tai chi on the death anxiety of elderly people living alone: the chain mediating effect of social support and psychological capital

**DOI:** 10.3389/fpsyg.2023.1303524

**Published:** 2024-01-08

**Authors:** Jiali Zhou, Baoyuan Wu, Lining Su, Xiujie Ma

**Affiliations:** ^1^School of Wushu, Chengdu Sports University, Chengdu, China; ^2^Clinical College, Hebei Medical University, Shijiazhuang, China; ^3^Chinese Guoshu Academy, Chengdu Sports University, Chengdu, China

**Keywords:** tai chi, death anxiety, elderly people living alone, social support, psychological capital

## Abstract

**Background:**

Population aging is a global trend, and the number of older adults living alone is increasing. Tai chi, a traditional Chinese exercise, has been shown to improve the physical and mental health of older adults.

**Aim:**

To investigate the effects of tai chi on death anxiety in older adults living alone and the role of social support and psychological capital in this relationship.

**Method:**

A cross-sectional study of 493 older adults living alone in four cities in southwestern China. Participants were assessed using questionnaires on tai chi practice, social support, psychological capital, and death anxiety.

**Results:**

Tai chi practice significantly reduced death anxiety in older adults living alone. It also positively correlated with social support and psychological capital, both of which negatively correlated with death anxiety. Social support and psychological capital mediated the relationship between tai chi practice and death anxiety, suggesting that tai chi may reduce death anxiety through these factors. These findings encourage older adults living alone to practice tai chi, as it may improve their mental and physical health and reduce their risk of death anxiety.

**Conclusion:**

Tai chi practice may reduce death anxiety in older adults living alone through the chain-mediated effects of social support and psychological capital. This suggests that tai chi may be a beneficial intervention for older adults living alone.

## 1 Introduction

Population aging has become a global phenomenon, and the degree of aging in China is also intensifying, with the number of elderly rising to 264 million by 2020, representing 18.7% of the total population (Online P. S. D, 2020). The China Development Research Foundation (CDRF) forecasts that China’s population aged 60 and older will approach 500 million by 2050, accounting for one-third of the country’s total population (Website C.N.S, 2020). In tandem with the development of Chinese families, there has been a marked increase in the number of empty nesters and elderly persons living alone (Wang and Zhao, 2015). Elderly adults living alone are individuals who do not live with their children and are unmarried, divorced, or widowed (Li et al., 2021). According to population estimates for the year 2020, the number of elderly individuals aged 65 and older living alone in China has reached 24.86 million, while the proportion of elderly individuals living alone has also increased (China N.B.O.S.O, 2021). According to studies, the unique living conditions and intergenerational contacts of older individuals living alone make them more likely to suffer negative emotions such as despair, anxiety, and loneliness than older persons living with their spouses or children (Li H. X. et al., 2019; Li Q. et al., 2019). They are a vulnerable segment of the geriatric population, and their mental health status is one of society’s most pressing needs for social care and assistance.

Moderate physical activity has a substantial positive effect on health interventions for older individuals who live alone. According to research, regular physical activity and exercise programs can assist enhance immunity (Scartoni et al., 2020), cardiovascular health (Vital et al., 2014), muscle strength (Vogel et al., 2009), balance (Barnett et al., 2003), and other factors in older persons. Furthermore, physical activity has been identified as an effective technique of reducing bad emotions and mental disease in older people, as well as assisting in the maintenance of cognitive functioning and enabling social relationships (Miller et al., 2019). The implementation of sustainable exercise programs is of utmost importance within the senior population residing independently, as it enables the fulfillment of their distinct physical requirements and health-related necessities (Byeon, 2019). Hence, physical exercise is regarded as a crucial intervention approach for enhancing the general health and quality of life among elderly individuals who reside in solitary conditions.

Several studies have revealed that tai chi, a traditional Chinese exercise program, has the potential to enhance and reduce negative psychological conditions, including anxiety, fear, and depression, among older persons. Following an extended period of structured and methodical tai chi practice, there was a notable decrease observed in the levels of state anxiety and trait anxiety among older individuals (Chang et al., 2013). Previous studies have included older persons exhibiting mild to moderate depressive symptoms as participants, and findings have demonstrated that engaging in regular tai chi training over an extended period can effectively decrease their depression scores (Liao et al., 2019). The extent of improvement in negative emotions such as depression, worry, and fear becomes increasingly evident with increased time of tai chi practice (Yan et al., 2013). Death anxiety is more common in elderly people, who have diminishing physical functioning, insecure economic and social support systems, and a reduced ability to care for themselves, all of which contribute to increased levels of death anxiety. An even more distinct subset of the elderly population consists of those who live alone (Bodner et al., 2015). Their absence of relatives, close friends, and other social supports makes them more susceptible to loneliness, which can exacerbate their death anxiety. Research on death anxiety among older persons has demonstrated a substantial correlation between physical condition and death fear (Missler et al., 2012). Specifically, older persons who are in good health exhibit lower levels of death anxiety (Moreno et al., 2009). As socioeconomic development continues, the improvement in people’s living standards has given attention to the mental health of elderly people living alone, and the aging of the population has emphasized the significance of promoting the mental health of elderly people living alone. Hence, the current study focused on the effects of tai chi, a traditional Chinese exercise, on death anxiety in elderly people living alone, as well as the inherent mechanisms of its effects, in order to provide a theoretical foundation for promoting the physical and mental health of elderly people living alone.

### 1.1 Tai chi exercise and death anxiety in the elderly living alone

Death anxiety is a fundamental tendency inherent in human beings that serves the purpose of self-preservation (Greenberg et al., 1986). The experience of anxiety and terror in response to mortality is attributed to the cognitive capacity to foresee the concept of the “inevitability of death” (Abeyta et al., 2014). Physical function deterioration, changes in living environment, and a reduction in social activities are all risk factors for unpleasant emotions such as despair, worry, and fear in the elderly (Moore et al., 2016; Soysal et al., 2017). Death is an unavoidable aspect of human existence, and when older people are exposed to more death cues due to the deaths of their peers and family, it increases their death anxiety. Some studies have shown that fear and anxiety about death increases with age and that death anxiety in older adults is a significant factor in their quality of life (Hui and Coleman, 2012). When compared to those who live with others, older persons who live alone are more likely to face mental health problems such as anxiety, loneliness, and panic; elderly people who live alone experience higher psychological distress and death dread. Researchers Missler et al. discovered a substantial correlation between physical health condition and death fear in an investigation of death anxiety in older persons (Missler et al., 2012). Furthermore, research suggests that physical activity helps older adults cope with death worry. Those who have been exercising regularly for a long period report feeling better about themselves and having less death dread (Liu et al., 2022).

Tai chi is one of the traditional Chinese physical activities, as well as one of the recommended activities in modern physical activities, and it serves a crucial role in promoting the physical and mental health of the elderly (Yang F. C. et al., 2022). Tai chi focuses attention through guided movement and thorough breathing, and seniors who engage in tai chi activities can achieve a harmonious balance of body and spirit. Numerous studies have demonstrated the benefits of tai chi for enhancing balance and muscle strength (Wehner et al., 2021) in the elderly, as well as its positive therapeutic and intervention effects on degenerative diseases (Oh et al., 2023), pain (Jones et al., 2012), metabolic disorders (Choi et al., 2017), and cardiovascular and cerebrovascular diseases (Yeh et al., 2009). In addition, tai chi has a substantial impact on the mental health of the elderly. Several studies have demonstrated that tai chi not only facilitates the improvement of cognitive function in the elderly, but it also improves the practitioner’s state of mind, decreases anxiety, and increases positive emotions while decreasing negative ones (Zhang et al., 2006). Some researchers have used the combination of tai chi and escitalopram to treat depression in the elderly, and the results confirm the effectiveness of tai chi as an adjunct treatment for depression in the elderly (Lavretsky et al., 2011). According to a 12-week study of older women, tai chi exercise enhanced their sleep quality, pain perception, and death dread (Bonab and Parvaneh, 2022). Some studies, in particular, have shown that tai chi practice, when compared to conventional medications, contributes to the reduction of anxiety in non-clinical populations and in patients with anxiety disorders (Wang et al., 2014), and that tai chi is suitable for elderly people to practice and significantly improves anxiety in the elderly (Song et al., 2014). The following study hypothesis is developed based on the preceding empirical findings:

H1: tai chi exercise has a negative effect on death anxiety in elderly people living alone.

### 1.2 Social support and its mediating effects

Social support encompasses the provisions of resources that an individual obtains by engagement in social activities, encompassing both tangible assistance and intangible assistance in the form of emotional or moral aid, derived from familial relationships, friendships, and other interpersonal connections (Wellman and Wortley, 1989). According to relevant studies, older adults who exercise have a high link with social support, and physical activity is one of the methods in which participants obtain social support (Da and Yao, 2012). Positive social support influences older persons’ physical activity and improves exercise outcomes (Resnick et al., 2002). To put it another, there exists a positive correlation between the level of social support available to older individuals and their likelihood of engaging in physical activity (Kouvonen et al., 2012).

In addition, numerous studies have demonstrated that social support facilitates senior adults’ ability to manage negative emotions (Rashedi et al., 2013). The greater the social support that elderly individuals receive, the fewer negative sentiments they will experience. Positive mental health is associated with high levels of social support, which is an effective method to alleviate negative emotions in older adults (Zhang and Zhou, 2022). Zhu’s study found a significant inverse relationship between depression and physical activity, but it also hypothesized that this relationship is most likely caused by the mediating effect of social support (Zhu et al., 2022). It also suggested that physical activity’s reach (location, peers, etc.) may be more significant in enhancing depressive mood than its volume (Mammen and Faulkner, 2013). It was also discovered in the study of social support and death anxiety in older persons that high levels of social support negatively predicted death anxiety in older adults. It was also discovered in the study of social support and death anxiety in older persons that high levels of social support adversely predicted death anxiety in older adults, with higher levels of social support being related with reduced death anxiety (Lim et al., 2017). Tai chi, as a group exercise, not only helps participants to have a pleasant and enjoyable activity experience while practicing, but it also widens the practitioner’s social network, allowing them to get greater social support (Ma et al., 2021). Therefore, we proposed the following hypotheses:

H2: Social support mediates the association between tai chi practice and death anxiety in older adults living alone.

### 1.3 Psychological capital and its mediating effects

Psychological capital is the positive psychological state of an individual that provides sufficient psychological resources for individuals to cope with negative external environments. Psychological capital is a crucial factor for individuals to successfully cope with crises and maintain mental health, and consists of four components: optimism, self-efficacy, and resilience (Avey et al., 2008). Previous research has found that physical exercise is strongly linked to psychological capital in older individuals, and that physical activity can alleviate negative emotions and boost good emotions in older adults, resulting in the buildup of psychological capital. Zhang discovered that positive mood indicators were influenced by one-time physical exercise while investigating the relationship between psychological capital and physical activity. Scholar Appelqvist discovered that with physical activity, a person’s subhealth progressively improves and positive psychological indicators rise to a degree (Appelqvist-Schmidlechner et al., 2017). Jiang study indicates that physical activity has positive effects on significant psychological distress in older adults (Jiang, 2019). Exercise increases good psychological feelings and psychological capital in older persons while decreasing depression, anxiety, and other negative emotions. In view of this, we proposed the following hypotheses:

H3: Psychological capital mediates the relationship between tai chi practice and death anxiety in older adults living alone.

### 1.4 Chain mediation of social support and psychological capital

Related research has found that social support and psychological capital may operate as intermediary variables in tai chi practice and death anxiety in elderly adults living alone (Qu et al., 2023; Zhao, 2023). Tai chi practice as a group exercise broadens social networks and provides additional social support during the participation process (Da and Yao, 2012). Social support for older persons has been shown to have a substantial impact on psychological capital; the more social support older adults receive, the more psychological capital they acquire (Guo and Liu, 2014). Furthermore, social support has the potential to indirectly impact death fear in older adults living alone by way of psychological capital. Higher psychological capital is associated with greater social support. Psychological capital has the potential to mitigate and ameliorate anxiety through enhanced regulation of individuals’ cognitive processes pertaining to mortality (Zhou et al., 2018). The findings of the study indicate that those who possess higher levels of psychological capital while engaging in physical exercise have reduced levels of death anxiety (Liu et al., 2022). Accordingly, the research hypothesis was formulated:

H4: Social support and psychological capital play a chain mediating role between tai chi practice and death anxiety of elderly living alone.

### 1.5 Hypotheses and conceptual model

**Figure 1:**
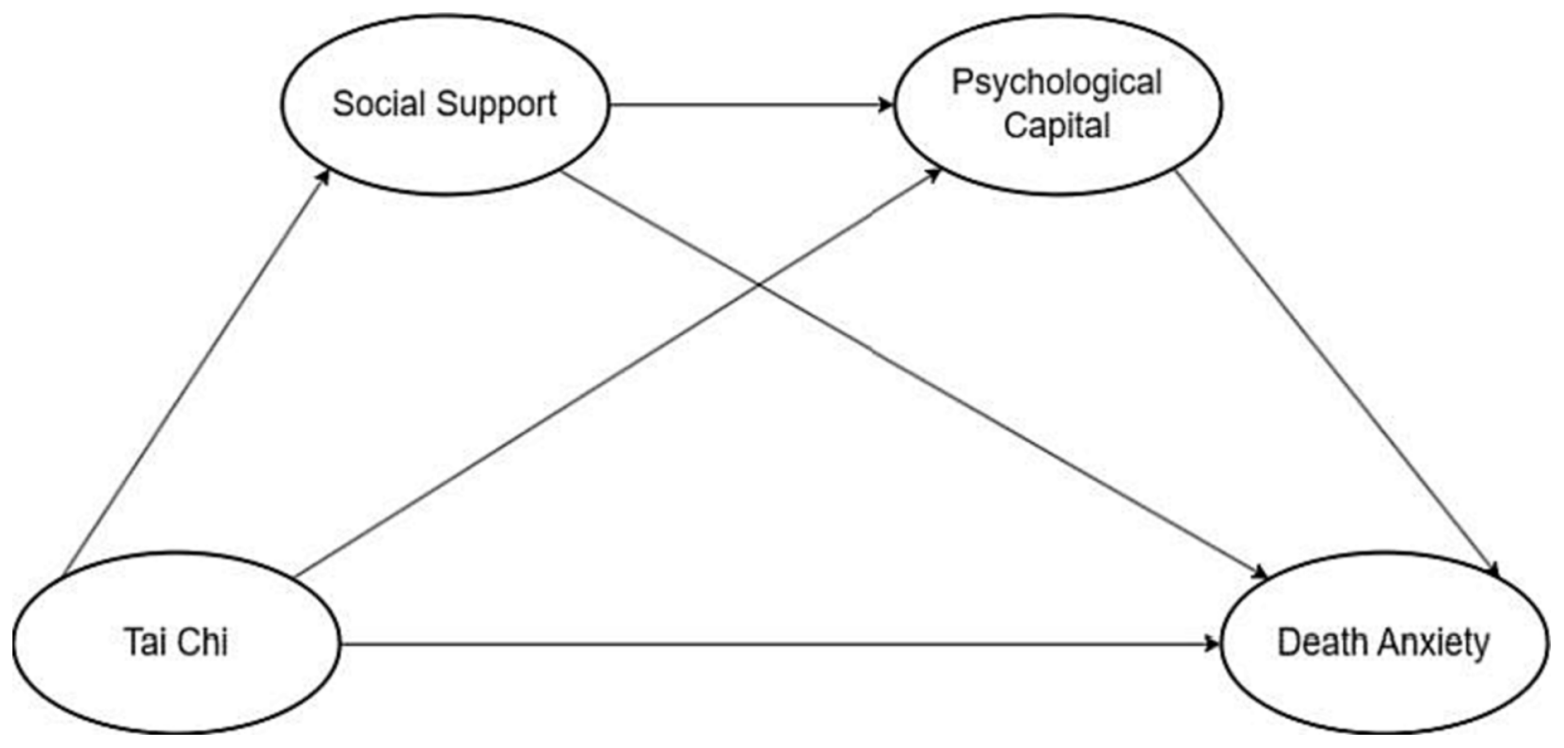
Hypothesized model of the effect of tai chi practice on death anxiety in elderly people living alone.

Overall, the literature and theoretical mechanisms discussed above suggest that practicing tai chi can provide multidimensional protective mechanisms for older adults, such as improving adverse emotions, increasing social support, and psychological capital. A multitude of studies have additionally illustrated the correlation between social support and psychological capital, as well as the manner in which individuals amass psychological capital via social support networks (Guo and Liu, 2014; Huang and Zhang, 2022). In previous studies, scholars investigated a variety of methods for modeling the effect mechanism of a certain exercise (Joseph et al., 2014; Yang Q. Q. et al., 2022; Qu et al., 2023), which serves as an important foundation for modeling in this work. However, because elderly people living alone are a marginalized group, it is necessary to investigate whether practicing tai chi can affect their death anxiety, as well as whether social support and psychological capital can play a mediating role in the relationship between practicing tai chi and death anxiety in elderly people living alone. Based on this, we constructed a research model. As shown in Figure 1, the model of this study shows that tai chi practice has a direct effect on death anxiety of elderly people living alone, and social support and psychological capital play a chain mediating role between tai chi practice and death anxiety of elderly people living alone.

## 2 Materials and methods

### 2.1 Participants and procedure

The questionnaires were distributed using the snowball sampling approach in this study. The sample in this study was rather unique because the respondents were older persons over 60 who lived alone. Beginning with a limited number of elderly samples from the general population, additional eligible samples were obtained through referrals from the surveyed samples to friends, relatives, neighbors, and leaders of social organizations for the elderly. The principal research areas were cities in southwest China, including Chengdu City, Sichuan Province, Chongqing City, Yunnan Province, and Guiyang City, Guizhou Province. The Chengdu Institute of Physical Education granted prior ethical sanction for the research. Before completing the questionnaire, the purpose of the survey and the intended use of the data were explicitly communicated, and anonymity, authenticity, and voluntariness were emphasized, with a response time of between 3 and 5 min. In the meantime, given the difficulty of completing the questionnaire for the elderly, the investigators could be tasked with completing the questionnaire on their behalf through verbal inquiry. As a token of appreciation, respondents were given a modest incentive of 3 RMB (approximately 0.42 cents) upon completion of the survey.

The survey will be conducted in April through July of 2023. The inclusion criteria for this study’s questionnaire were (1) elderly persons aged 60 and older who lived alone, (2) who participated in tai chi exercise, (3) provided informed consent, and (4) did not have any major physical or mental problems. Exclusion criteria for invalid questionnaires were (1) repeated invalid questionnaires and (2) questionnaires that took less than 1 min to complete. A total of 550 questionnaires were distributed, and 493 were returned, yielding a 90% validity rate. The suitability of the sample size for this study was assessed using Li H. X. et al. (2019) sample size calculation method, G*Power 3.1. The post-hoc statistical efficacy test (effect size = 0.25, *α* = 0.05) revealed that power = 1, indicating that the sample size is sufficient (Table 1).

**Table 1:** Participant demographics.

Demographic category	Frequency	Percent (%)
Gender		
Male	243	49.3
Female	250	50.7
Age		
60–64	229	46.5
65–69	205	41.6
70–74	24	4.9
75–79	27	5.5
80 and above	8	1.6
Educational level		
Primary school and below	138	28.0
middle school	74	15.0
High school or technical secondary school	83	16.8
College (including higher vocational education)	65	13.2
Bachelor degree or above	133	27.0
Marital status		
single	176	35.7
Separation or divorce	160	32.5
widowhood	157	31.8
Monthly income		
1,500 and below	121	24.5
1,501–3,500	125	25.4
3,501–5,000	132	26.8
5001 and above	115	23.3

### 2.2 Instruments

#### 2.2.1 Tai chi exercise level

Based on Liang’s (Liang, 1994) Physical Activity Rating Scale (PARS-3), the exercise level of tai chi practitioners was quantitatively evaluated in this study by measuring intensity, time, and frequency. Each indicator is categorized into five grades, of which duration, intensity and frequency are graded from 1 to 5 and scored from 1 to 5. Follow Liang’s measurement formula to obtain the investigator’s tai chi practice: tai chi activity = intensity × (time-1) × frequency, with a total score ranging from 0 to 100 points, and the higher the score the higher the level of tai chi practice.

Tai chi Frequency: 1 point for “tai chi practiced 0–1 times per month on average, “2 points for “tai chi practiced 2–3 times per month on average, “3 points for “tai chi practiced 1–2 times per week on average, “4 points for “tai chi practiced 3–5 times per week on average, “and 5 points for “tai chi practiced 1 time per day on average.”

Tai chi time: “average practice of tai chi for less than 10 min” scored 1 point, “average practice of tai chi for 10–20 min” scored 2 points, “average practice of tai chi for 20–30 min” scored 3 points, “average practice of tai chi for 30–60 min” scored 4 points, and “average practice of tai chi for 60 min and above” scored 5 points. 3 points for “an average of 30 to 60 min of tai chi,” 4 points for “an average of 60 min or more of tai chi,” and 5 points for “an average of 10 to 20 min of tai chi.”

Tai chi intensity: “Average practice of tai chi almost no heat” score 1 points, “average practice of tai chi body slightly hot but no sweat” score 2 points, “average practice of tai chi body slightly sweat” score 3 points, “average practice of tai chi body sweat more” score 4 points, “average practice of tai chi body sweat” score 5 points.

Cronbach’s alpha for this scale was 0.843.

#### 2.2.2 Social support

We used a social support scale developed by Zimet et al. (1988). The scale primarily assesses the level of social support that people feel from a variety of sources. It is comprised of three dimensions: support from friends and family, support from other people (such as leaders, coworkers, classmates, relatives, etc.), and total score, which represents the total amount of social support that people feel. The subject experiences and receives more social support the higher their overall score. The measure performs well in surveys of Chinese older persons in terms of validity and reliability (Pang et al., 2019). “My family is able to help me in a practical and concrete way” is one of the 12 self-assessment items on the scale. The items are rated on a 5-point Likert scale, which goes from Strongly Disagree to Strongly Agree. The ratings are assigned a value of 1 to 5, which is descending. Cronbach’s alpha for this scale was 0.843.

#### 2.2.3 Psychological capital

We used Zhang et al. (2010) scale to measure the psychological capital of elderly people living alone. There are four components to it: resilience, self-efficacy, optimism, and hope. There were 26 questions in all, divided into 6 items for optimism, 6 items for hope, 7 items for self-efficacy, and 7 items for resilience. A 5-point Likert scale with five levels—from strongly disagree to strongly agree—was used to rate the question items, and the results were awarded a score between 1 and 5 in that order. Stronger scores indicated stronger positive inclinations in psychological capital, whereas lower scores indicated higher negative tendencies. Greater scores represent greater positive tendencies in psychological capital, whereas lower scores indicate larger negative tendencies when taking into account the dimension scores and the total score. Cronbach’s alpha for this scale was 0.91.

#### 2.2.4 Death anxiety

The Death Anxiety Scale grading system is based on the Prof. Templer (1970) Death Anxiety Scale, which was created in 1967. The scale is a unidimensional scale containing 15 entries. Nine of the entries are positively scored and the remaining six are negatively scored. The scale has been widely utilized in research of Chinese older persons because it has good measurement qualities in the context of Chinese culture (Yang et al., 2013). A 5-point Likert scale with five levels—from strongly disagree to strongly agree—was used to grade the questions, and each level was given a score between 1 and 5 in that order. Subjects with higher scores on the Death Anxiety Scale had higher degrees of death anxiety. Cronbach’s alpha for this scale was 0.957.

## 3 Results

### 3.1 Reliability and validity tests

Because the questionnaire for this study was built using past study questionnaires, the scale’s reliability and validity required to be confirmed. To establish the convergent validity of all constructs, a validated factor analysis (CFA) was performed first, followed by additional computations of average variance extracted and combined reliability. Table 2 shows that the convergent validity (AVE) of all items was greater than 0.5 and the component reliability (CR) of all items was greater than 0.7, indicating that the test criteria were met (Zhou and Long, 2004). Therefore, the analyzed data have good aggregation (convergent) validity.

**Table 2:** validity and reliability test of the questionnaires.

Variable	AVE	CR
TC	0.643	0.844
SS	0.556	0.790
PC	0.567	0.838
DA	0.544	0.827

CR, composite reliability; AVE, average variance extracted; TC, tai chi; SS,social support; PC, psychological capital; DA, death anxiety.

### 3.2 Descriptive statistics and correlations between the main study variables

Table 3 displays the means, standard deviations, and correlation coefficients of the primary research variables. Table 3 illustrates how correlations were utilized to examine the relationships between a total of four items: death fear, psychological capital, social support, and tai chi exercise. Pearson correlation coefficients were employed to assess the strength of the relationships. Specific analyses showed that: tai chi practice was positively correlated with social support (*r* = 0.508, *p* < 0.01), there was a positive correlation between tai chi practice and psychological capital (*r* = 0.501, *p* < 0.01), and there was a negative correlation between tai chi practice and death anxiety (*r* = −0.436, *p* < 0.01); there was a positive correlation between social support and psychological capital (*r* = 0.478, *p* < 0.01), there was a negative correlation between social support and death anxiety (*r* = −0.442, *p* < 0.01); and there was a negative correlation between psychological capital and death anxiety (*r* = −0.433, *p* < 0.01). The presence of associations among the aforementioned factors offers preliminary evidence in favor of the hypotheses proposed in this research.

**Table 3:** Descriptive statistics and correlations for primary variables.

Variable	M	S.D.	TC	SS	PC	DA
TC	3.1684	1.07753	1			
SS	3.0673	0.89992	0.508**	1		
PC	3.0971	0.85302	0.501**	0.478**	1	
DA	2.9279	1.02426	−0.436**	−0.442**	−0.463**	1

*n* = 493; M, mean; S.D., standard deviation.

*p < 0.05.

**p < 0.01.

### 3.3 Mediating analysis

The current study employed structural equation modeling to conduct a chained mediation effect test in order to effectively control measurement error and assess the mediating roles of social support and psychological capital between tai chi practice and death anxiety. AMOS24. 0 was used to examine the association between tai chi practice, social support, psychological capital, and death anxiety using Marsh et al. (2014) proposed mediation effect test process. Table 4 displays standard findings for the fitting indices, and the model parameters meet the fitting requirements.

**Table 4:** Model fit.

	CMIN/DF	GFI	AGFI	RMSEA	NFI	TLI	CFI	RFI	IFI
Model	1.301	0.947	0.936	0.025	0.955	0.988	0.989	0.950	0.989

CMIN/DF, chi-square ratio of degrees of freedom; GFI, goodness-of-fit index; AGFI, adjusted goodness-of-fit index; RMSEA, root mean square error of approximation; NFI, normative fit index; IFI, increased fit index; CFI, comparative fit index; RFI, relative fit index; IFI, value-added fit index.

Using the bias-corrected non-parametric percentile Bootstrap approach, the significance of individual mediating effects was assessed in order to confirm the mediating role of psychological capital and social support. Hayes proposed that the number of replicate samples of the original sample in the bootstrap mediation effect test be at least 1,000 (Hayes, 2009). We determine the 95% confidence interval (CI) by running the mediation effect test with a bootstrap sample size of 5,000. If the standardized path coefficients of the 95% CI do not contain 0, then the mediation effect is significant. As can be seen from Table 5, the direct effect of tai chi practice on death anxiety in elderly people living alone was significant (direct effect = −0.179, 95% CI [−0.325, −0.041]). Indirect effects contained 3 significant mediating pathways: tai chi practice → social support → death anxiety (indirect effect = −0.132, 95% CI [−0.259, −0.037]); tai chi practice → psychological capital → death anxiety (indirect effect = −0.084, 95% CI [−0.179, −0.026]); tai chi practice → social support → psychological capital → death anxiety (indirect effect = −0.061, 95% CI [−0.125, −0.024]).

**Table 5:** Test results of mediation effects.

effect	Parameter	Estimate	Bootstrap LLCI	Bootstrap ULCI
Indirect effect	TC → SS → DA	−0.132	−0.259	−0.037
	TC → PC → DA	−0.084	−0.179	−0.026
	TC → SS → PC → DA	−0.061	−0.125	−0.024
Direct effect	TC → DA	−0.179	−0.325	−0.041
Total effect	TC → SS → PC → DA	−0.457	−0.565	−0.352

The Bootstrap sample size is set at 5000.

**Figure 2:**
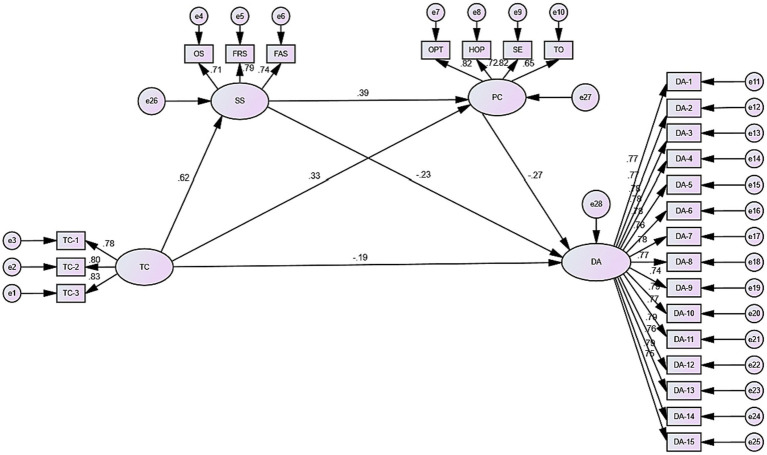
Intermediary model diagram.

Furthermore, the standardized path coefficient model of tai chi practice affecting death anxiety is shown in the figure: the standardized path coefficient was significant (*β* = −0.190, *p* < 0.001) when tai chi practice → death anxiety was affected, thus indicating that tai chi practice exerts a significant negative influence relationship on death anxiety. That is, hypothesis H1 was established. The path coefficient of tai chi practice → social support (*β* = 0.62, *p* < 0.001) → death anxiety (*β* = −0.23, *p* < 0.001) was significant, indicating that social support has a mediating role between tai chi practice and death anxiety. That is, hypothesis H2 was established. The path coefficient of tai chi practice → psychological capital (*β* = 0.33, *p* < 0.001) → death anxiety (*β* = −0.27, *p* < 0.001) was significant, indicating that psychological capital mediates the relationship between tai chi practice and death anxiety. That is, hypothesis H3 was established. The path coefficient of tai chi practice → social support → psychological capital (*β* = −0.27, *p* < 0.001) → death anxiety was significant, indicating that social support and psychological capital have a chain-mediated role between tai chi and death anxiety. That is, hypothesis H4 was established (Figure 2).

## 4 Discussion

### 4.1 Direct effect of tai chi practice on death anxiety in elderly people living alone

The findings of this study imply that continuing to practice tai chi can reduce death anxiety in older adults who live alone, improve the quality of elderly people’s life, and support their physical and mental health development, which is consistent with prior research (Song et al., 2014; Wang et al., 2014; Yang F. C. et al., 2022). Tai chi is a traditional Chinese exercise regimen that emphasizes softness and the harmony of yin and yang. Its features include slow, deliberate motions, abdominal breathing, and energy focus (Zhang et al., 2022). Tai chi controls the functioning of the hypothalamic–pituitary-adrenergic axis, particularly by reducing stress-related adrenocorticotropic hormone, dehydroepiandrosterone, and other hormones, hence alleviating negative feelings like depression and anxiety (Cho, 2008). Tai chi training also includes Chinese medicine notions such as qi, blood, fluid, and meridians to help participants with anxiety and despair (Jiang et al., 2006). These findings support the neurobiological concept as well. The neurobiological hypothesis proposes that physical activity may alleviate depression in older adults by slowing the HPA axis’s response to stress, or that increasing the brain-derived neurotrophin factor (BDNF) may alleviate anxiety by slowing hippocampal atrophy and increasing an individual’s positive feelings (Erickson et al., 2012). This explanation has the positive conclusion that elderly people living alone can enhance their physiological mechanisms to withstand death anxiety by practicing tai chi. Furthermore, tai chi also emphasizes the “Yi Lian” of “although the appearance is slightly slowed, but the inner meaning cannot be stopped, “which focuses the exerciser’s attention on the boxing frame, strengthens the brain’s sensitivity to the movement itself, eliminates attention to other external stimuli, and thus focuses on the completion of the movement and concentration of the idea (Qian, 2008). This also coincides with Western research on the “Attention Shift Hypothesis, “(Breus and O'Connor, 1998) which postulates that elderly single people can generate positive emotional “foci of excitement” to counteract death anxiety by practicing tai chi and separating themselves from the state of death anxiety. Furthermore, the link between physical exercise and death anxiety has been verified in recent papers in the context of COVID-19 investigations. Because the senior population is more vulnerable to COVID-19 during the pandemic, older persons living alone have more intense death anxiety (Khademi et al., 2021), and physical activity is one of their key coping mechanisms (Han, 2019). It may be demonstrated that the benefits of tai chi exercise can reduce death anxiety in older people who live alone.

### 4.2 Mediating role of the social support in the influence of tai chi on death anxiety in older adults living alone

The results of this study demonstrated that tai chi practice positively predicted social support (*β* = 0.63, *p* < 0.001), social support negatively predicted death anxiety in older adults living alone (*β* = −0.23, *p* < 0.001), and that social support played a mediating role between tai chi practice and death anxiety in older adults living alone. Social support relieves stress and increases pleasant emotions, which enhances physical and mental health in the elderly (Zhu et al., 2022). Studies have indicated a positive correlation between death anxiety and social support among physical activity participants. This is because engaging in physical activity is a key strategy for overcoming negative emotions like anxiety, depression, and loneliness, and social support is typically used as a gage for older adults’ quality of life and overall wellbeing (Liu et al., 2022). The results of a four-month clinical trial show that elderly people who are depressed, nervous, or have little social support can benefit from physical activity in terms of their emotional condition, degree of social support, and overall quality of life (Ruiz-Comellas et al., 2022). A comprehensive review also found that socially supported older people were more likely to actively participate in amateur sports activities. This data, on the other hand, underscores the critical importance of social support in encouraging older people to participate in physical activity (Smith et al., 2017). Theoretically, this study is supported by the Wang and Ebrahimi study, which indicates that social support is critical for the development of older adults’ physical and mental health and that negative psychological emotions, such as death anxiety, are less common in those with greater social support (Ebrahimi et al., 2018; Wang et al., 2021). Another study found that older adults can gain more social support through interpersonal interactions in sports that build or improve the structure of an individual’s social network (Cohen et al., 2000), which can positively influence an individual’s assessment of his or her ability to cope with stressful situations and provide the individual with direct access to resources for stress resolution (Kawachi and Berkman, 2001). As a result of this research, we may conclude that social support has a considerable effect on death anxiety in older adults who live alone. Against the backdrop of shrinking family sizes and decreasing intergenerational exchanges, the elderly living alone promoted interpersonal interactions and gained more social support while exercising by participating in tai chi practice and establishing new social networks with like-minded people; however, the high level of social support negatively affected the elderly living alone’s death anxiety. This supports the link between social support and tai chi practice and death anxiety in older people who live alone.

### 4.3 Mediating role of the psychological capital in the influence of tai chi on death anxiety in older adults living alone

The findings indicated that tai chi practice positively predicted psychological capital (*β* = 0.33 *p* < 0.001), psychological capital negatively predicted death anxiety in older adults living alone (*β* = −0.27 *p* < 0.001), and psychological capital mediated the relationship between tai chi practice and death anxiety in older adults living alone. The findings of this study are consistent with prior research that has shown that exercise can successfully reduce feelings of low self-esteem in college students, increase physical self-esteem and psychological capital, and improve mental health (Qin and Yuan, 2019). Some scholars have also noted a significant correlation between physical activity and psychological capital of rural empty nesters, and that the psychological capital status of the elderly changes with their level of physical activity, with the higher the level of physical activity, the higher the psychological capital of the elderly (Ou, 2021). Furthermore, physical activity and psychological capital have a significant impact on people’s mental health (Ishikawa et al., 2006). Psychological capital is positively associated with and reduces death anxiety in elderly people (Sharma et al., 2019).

The mediating role of psychological capital in tai chi practice and death anxiety among elderly people living alone is demonstrated by the significant correlation between the dimensions of psychological capital and them. Psychological capital, a positive psychological quality comprised of the four dimensions of self-efficacy, resilience, sense of hope, and optimism, plays an essential role in elderly adults’ responses to negative emotions (e.g., depression, anxiety). In particular, psychological capital’s self-efficacy can support older adults living alone in discovering their own value through physical activity, which boosts their self-esteem and pleasure perception (Lin et al., 2022). In order to improve psychological resilience, resilience in psychological capital can assist elderly people living alone in building up psychological resources through physical activity. This will enable them to bounce back quickly from setbacks when confronted with unfavorable information, such as reminders of impending death (Xin and Ma, 2023). The psychological capital sense of hope also assists the elderly living alone to maintain physical health through tai chi exercise, lessen fear and worry about death and future life, and develop optimism for the future (Peh et al., 2017). Optimistic qualities in psychological capital have been identified as one of the key personality elements influencing anxiety in older persons (Dolcos et al., 2016). As a result of the findings of this study, elderly people living alone boosted their psychological capital through tai chi practice, which not only improved their physical health but also helped to lessen their negative emotions and death anxiety.

### 4.4 Chain mediating role of the social support and psychological capital in the effect of tai chi on death anxiety In older adults living alone

Tai chi practice’s main avenue to reducing death concern in older individuals living alone is through social and psychological consequences. Through tai chi practice, elderly people living alone strengthen social network connections between individuals and between individuals and groups, which improves the level of social support in order to increase the level of psychological capital, reducing the death anxiety of elderly people living alone. Previous study findings complement the conclusions of this mediation model, with Rabenu indicating that persons with access to social support will be able to use their high psychological capital to cope with stress and adapt to volatile situations (Rabenu et al., 2017). Another study, this one with emergency room physicians as subjects, indicated that psychological capital can help with depression by enhancing social support (Xu et al., 2022). According to Huang’s findings, persons who received more social support during the COVID-19 pandemic had more psychological capital, allowing them to cope with the stress and anxiety of pandemic uncertainty and fear (Huang and Zhang, 2022). As a conclusion, the present study is consistent with the above studies and further validates the relationship between social support and psychological capital.

One of the key tenets of social capital theory is social support (Hou, 2001). According to this theory, older people who engage in physical activity with one another socially are building their social capital and gaining social support (material or emotional). Additionally, according to social network theory, older people who participate in social activities are able to receive a certain amount of official or informal social support, which has a protective effect on their mental health as they age (Liang and Jia, 2022). Stated differently, social support plays a critical role in the development of older adults’ physical and mental health and is strongly correlated with their engagement in sports. According to certain research, older persons who experience excessive life stress and a lack of social support are more likely to develop mental diseases such as depression and anxiety (Aneshensel and Stone, 1982). Physical exercise prevents or improves depressed mood in old age by meeting older people’s basic psychological needs (i.e., maintaining or reconfiguring social connections to meet relational needs and obtaining health-related information, resources, and emotional attachments to meet autonomy needs), which improves geriatric mental health (Zhu et al., 2022). Tai chi practice, as a group fitness program, can help the elderly broaden their social network and increase their social support. Participating in tai chi practice improves the social participation level of elderly people living alone, provides new social support, and provides information and emotional support, which increases the level of positive psychological capital and thus improves the mental health of elderly people living alone. Simultaneously, social support services aided the elderly living alone in a variety of ways, including increasing their psychological capital to cope with negative information such as death reminders and decreasing dread of death anxiety among the elderly living alone. The study’s findings also represent the virtuous double circle of “individual-society” and “physical health-mental health, “emphasizing the connection between the elderly living alone and society, as well as the reciprocal relationship between physical and mental health.

## 5 Limitations and influences

This study contains a number of restrictions. Firstly, the cross-sectional nature of this study made it impossible to establish a causal association between the variables; therefore, a longitudinal intervention experiment will be required to look into this relationship in the future. Secondly, the study did not completely elucidate the processes by which practicing tai chi reduces death anxiety in elderly persons living alone; instead, it focused solely on the mediating effects of psychological capital and social support on tai chi and death anxiety in elderly people living alone. Thirdly, the sample distribution is small, so the research conclusions may have a geographic bias. However, the research conclusions can be improved in the future by expanding the scope of the collection region. The survey respondents are a group of old people living alone in southwest China. Lastly, the survey population in this study consisted of people over 60 who were living alone. They were not separated into low, middle, or high old age groups. Future research can examine whether these three categories of older people are equally suitable for this research model and whether the pathway mechanism of tai chi practice affecting the improvement of death anxiety is consistent.

Despite these limitations, the research has theoretical and practical ramifications. Firstly, this study investigated the relationship between tai chi practice and death anxiety in older adults living alone, broadening research and theoretical knowledge about physical activity and death anxiety in older adults. Secondly, the current study broadens the study of death worry; earlier, researchers linked people’s anxiety over death’s moderating function to worldview defenses (Dechesne et al., 2000). This study examined the relationship between tai chi practice and death anxiety and discovered that both death anxiety and social support might have a chain-mediated effect. This opens up new avenues for investigation and also offers a theoretical foundation for the mental health of the elderly. Thirdly, research indicates that practicing tai chi lowers death anxiety. This discovery should motivate seniors who live alone to practice the art form, as it not only enhances physical and mental well-being but also lessens the fear of dying and increases old age well-being. Fourth, the favorable benefits of tai chi on death anxiety in elderly adults living alone provide healthcare practitioners with important practical value. Tai chi can be incorporated into rehabilitation programs for elderly people living alone as a comprehensive, low-risk therapy, and clinical practitioners can recommend tai chi as a nonpharmacological adjunctive therapy in treatment protocols to improve patients’ psychological and physical well-being.

## 6 Conclusion

This study shows that there is a significant correlation between tai chi practice, social support, psychological capital and death anxiety of elderly people living alone, and tai chi practice has a significant negative predictive effect on death anxiety of elderly people living alone, and tai chi practice can directly affect death anxiety of elderly people living alone as well as indirectly affect death anxiety of elderly people living alone through the intermediary effects of social support and psychological capital. More specifically, there are three ways that tai chi practice reduces death anxiety in older adults who live alone: via the mediated effect pathway of psychological capital, through the mediated effect pathway of social support, and through the mediated effect pathway that runs through the combination of psychological capital and social support. The current study offers new insights on coping with mortality fear in older persons, with the goal of improving the well-being of older adults who live alone.

## Data availability statement

The data presented in this study are available upon request from the corresponding author. The data are not publicly available due to an ethical agreement with the Chengdu Sport University Social Sciences Ethics Panel.

## Ethics statement

The studies involving humans were approved by the Ethics Committee of Chengdu Sport University (code 2022-85, approved 15/08/22). The studies were conducted in accordance with the local legislation and institutional requirements. Written informed consent for participation in this study was provided by the participants' legal guardians/next of kin.

## Author contributions

JZ: Data curation, Formal analysis, Investigation, Methodology, Resources, Software, Visualization, Writing – original draft, Writing – review & editing. BW: Funding acquisition, Investigation, Methodology,Writing – review & editing. LS: Funding acquisition, Investigation, Resources, Visualization, Writing – review & editing. XM: Conceptualization, Data curation, Funding acquisition, Investigation, Methodology, Project administration, Resources, Software, Supervision, Validation, Visualization, Writing – original draft, Writing – review & editing.
